# Discordance between CECT and angiographic findings in severe acute pancreatitis-related hemorrhage: implications for interventional management

**DOI:** 10.3389/fmed.2026.1845142

**Published:** 2026-06-01

**Authors:** Yinshan Wu, Jiannan Qian, Like Qian, Li Chen, Bo Shen, Feng Guo

**Affiliations:** 1 Department of Critical Care Medicine, School of Medicine, Sir Run Run Shaw Hospital, Zhejiang University, Hangzhou, Zhejiang, China; 2 Department of Radiology, School of Medicine, Sir Run Run Shaw Hospital, Zhejiang University, Hangzhou, Zhejiang, China; 3 Department of General Surgery, School of Medicine, Sir Run Run Shaw Hospital, Zhejiang University, Hangzhou, Zhejiang, China

**Keywords:** clinical decision-making, contrast-enhanced CT, digital subtraction angiography, hemorrhage, pseudoaneurysm, severe acute pancreatitis, transcatheter arterial embolization

## Abstract

**Background:**

Hemorrhagic complications in severe acute pancreatitis (SAP) carry high mortality and demand timely intervention. Contrast-enhanced computed tomography (CECT) is routinely performed before digital subtraction angiography (DSA) to guide management decisions, yet its predictive accuracy for angiographic findings and interventional success remains poorly defined.

**Materials and methods:**

We conducted a retrospective analysis of 69 bleeding episodes in 56 SAP patients with clinical suspicion of active hemorrhage who underwent both CECT and subsequent DSA. Imaging findings were graded on a 3-point scale (Grade 0: negative; Grade 1: suspicious; Grade 2: definite bleeding). Intermodality concordance was evaluated using weighted kappa statistics, with DSA serving as the reference standard. Clinical outcomes including embolization success, rebleeding, and need for surgical rescue were recorded.

**Results:**

The intermodality agreement between CECT and DSA was fair (weighted κ = 0.29). Among 26 episodes with negative CECT findings (Grade 0), 69.2% (18/26) required and successfully underwent therapeutic embolization followed by clinical stabilization. Although CECT Grade 2 findings showed a high positive predictive value (94.1%), they were also associated with all instances of rebleeding and procedural abandonment. Nine episodes eventually required surgical rescue after DSA.

**Conclusion:**

In this selected high-risk cohort of SAP patients undergoing both CECT and DSA for clinically suspected hemorrhagic complications, CECT demonstrated only limited concordance with angiographic findings. Negative or non-definitive CT findings should be interpreted cautiously when clinical suspicion for ongoing hemorrhage persists, and angiographic evaluation should be considered as part of an integrated, clinically driven diagnostic and therapeutic pathway in this high-risk population.

## Introduction

Severe acute pancreatitis (SAP) has a mortality rate of 15–30%, and major hemorrhagic complications are among the most acutely life-threatening events in its course (1–3). These vascular complications, such as pseudoaneurysm formation, arterial erosion, and active extravasation, result from the proteolytic and inflammatory destruction of peripancreatic and intrapancreatic vessel walls by activated pancreatic enzymes like elastase and trypsin (4, 5). The reported incidence of clinically significant hemorrhage in SAP ranges from 1.2 to 14.5%; however, when it occurs, the associated mortality exceeds 30% without timely intervention (6, 7).

Major hemorrhage in SAP may manifest through diverse clinical presentations: intraperitoneal bleeding, retroperitoneal hemorrhage, gastrointestinal bleeding via enteric fistulas (8), bleeding into pseudocysts or walled-off necrosis cavities, or external hemorrhage through percutaneous drainage tracts (9). The clinical management of these complications hinges on the rapid identification of the bleeding source and timely intervention.

Timely diagnosis and intervention are critical for patient survival in such cases. Currently, contrast-enhanced computed tomography (CECT) is the first-line imaging modality for evaluating SAP, providing crucial information regarding the extent of necrosis and the presence of local complications (10, 11). In the setting of suspected hemorrhage, CECT is widely utilized to identify signs of bleeding, such as contrast extravasation, hematoma expansion, or pseudoaneurysm formation, thereby acting as a “gatekeeper” to determine which patients require catheter-based angiography.

However, the reliability of CECT in excluding active hemorrhage remains debatable. Although CECT is excellent for anatomical localization, it has documented limitations in detecting intermittent arterial bleeding, venous bleeding, or hemorrhage obscured by complex necrotic collections (1). Digital subtraction angiography (DSA) is widely regarded as the reference standard for diagnosing arterial vascular lesions and offers the additional advantage of immediate therapeutic embolization (5, 6). Recent data suggest that relying solely on CECT may lead to missed diagnoses; a study by Mohamed et al. reported a false-negative rate of 16.1% for CECT in detecting visceral artery pseudoaneurysms compared with angiography (12). Furthermore, in hemodynamically unstable patients or those with obscure bleeding sources, the sensitivity of noninvasive imaging may be insufficient to rule out the need for intervention (13).

Accurate radiological assessment is paramount as delayed identification of SAP-associated hemorrhage directly correlates with poor outcomes. Therefore, this study aims to quantify the diagnostic gap between CECT and DSA and to evaluate its implications for clinical decision-making and intervention timing.

## Methods

### Study design and population

This retrospective study evaluated the diagnostic performance of CECT relative to that of digital subtraction angiography in SAP-associated hemorrhagic complications. The study was conducted in accordance with the Declaration of Helsinki and approved by the Institutional Ethics Committee of Sir Run Run Shaw Hospital (20262019). Adult patients diagnosed with SAP who experienced sudden life-threatening hemorrhagic events between January 2015 and January 2025 were identified through a systematic screening of the institutional electronic medical record (EMR) system (14, 15).

### Data quality control and manual audit

A two-stage manual audit process was implemented to improve the case ascertainment and data reliability. In the first stage, potential cases were identified using automated EMR queries based on diagnostic codes, interventional records, and documented surgical procedures. In the second stage, two independent clinicians manually reviewed all candidate cases by examining the admission notes, daily progress notes, interventional radiology (IR) records, and operative reports.

Patients were excluded according to the following criteria.

(1) A primary diagnosis other than SAP (e.g., chronic pancreatitis or nonpancreatic etiologies).
(2) Digital subtraction angiography (DSA) was performed for indications unrelated to hemorrhagic complications.
(3) Substantial absence of core clinical, imaging, or interventional data.

Discrepancies identified during the manual review were resolved through consensus discussions with two senior attending physicians.

### Subgroup definition and imaging evaluation

For this analysis, a specific subgroup was established consisting of patients who underwent both CECT and DSA during the same period. All CECT examinations were performed using a standardized multiphasic abdominal enhancement protocol, including non-contrast, arterial phase (25–30 s), and portal venous phase (65–70 s) acquisitions following intravenous contrast administration (1.5 mL/kg; injection rate 3 mL/s). Routine arterial-phase image reconstruction thickness was 5 mm, with thinner 2 mm reconstruction performed when clinically indicated.

To standardize the evaluation of imaging findings, the following classification systems were used for CT and DSA reports:

CT bleeding likelihood classification: Grade 0 (Negative), no vascular abnormality or hematoma expansion; Grade 1 (Suspicious), suspicious high-density foci or vascular irregularity; Grade 2 (Definite), clear contrast extravasation or pseudoaneurysm.

DSA diagnostic classification: Grade 0 (Negative), no signs of hemorrhage; Grade 1 (Indirect), vascular irregularity, suspicious vasospasm, or suspicious parenchymal staining; Grade 2 (Definite), contrast extravasation, pseudoaneurysm, or definitive identification of the culprit vessel.

DSA intervention and outcome assessment: DSA outcomes were categorized into five groups based on angiographic findings and clinical status within 48 h post-procedure. Result 0 (Neg-Active), negative angiography with clinically suspected persistent hemorrhage; Result 1 (Embo-Success), successful control via embolization; Result 2 (Embo-Rebleed), rebleeding after technical success; Result 3 (Neg-Stable), negative findings with subsequent clinical stabilization; Result 4 (Abandoned), procedure terminated due to instability.

### Data collection and variable definitions

Demographic and clinical variables were extracted from the validated EMR database using standardized case report forms. Baseline characteristics included age, sex, and the etiology of SAP. Hemorrhage-related variables included the interval from SAP onset to the hemorrhagic event (days from onset to bleeding), the total number of hemorrhagic episodes during hospitalization (bleed count), and the presence of infected peripancreatic necrosis (IPN) at the time of bleeding. Previous interventions before the hemorrhagic event, including percutaneous catheter drainage (PCD) and surgical necrosectomy, were recorded. Clinical presentations at the time of bleeding were categorized into six standard types: (1) bloody drainage, (2) decrease in hemoglobin level, (3) abdominal pain, (4) gastrointestinal bleeding, (5) detected on routine CECT follow-up, and (6) combined drainage and gastrointestinal bleeding. The primary outcome was in-hospital mortality, defined as death during the index hospitalization.

### Statistical analysis

The study population was characterized at two levels: the patient level (*N* = 56) for baseline demographics and clinical outcomes and the bleeding-event level (*n* = 69) for imaging findings and diagnostic performance. Continuous variables were expressed as medians with interquartile ranges (IQRs), and categorical variables were presented as frequencies and percentages. The intermodality agreement between CECT and DSA grading was assessed using weighted Cohen κ statistics with quadratic weights (16). Diagnostic performance of CECT was calculated using DSA as the reference standard under three predefined positivity thresholds. Sensitivity, specificity, positive predictive value, and negative predictive value were calculated at the bleeding-event level. All statistical analyses were performed using R software (version 4.5).

## Results

**Figure 1:**
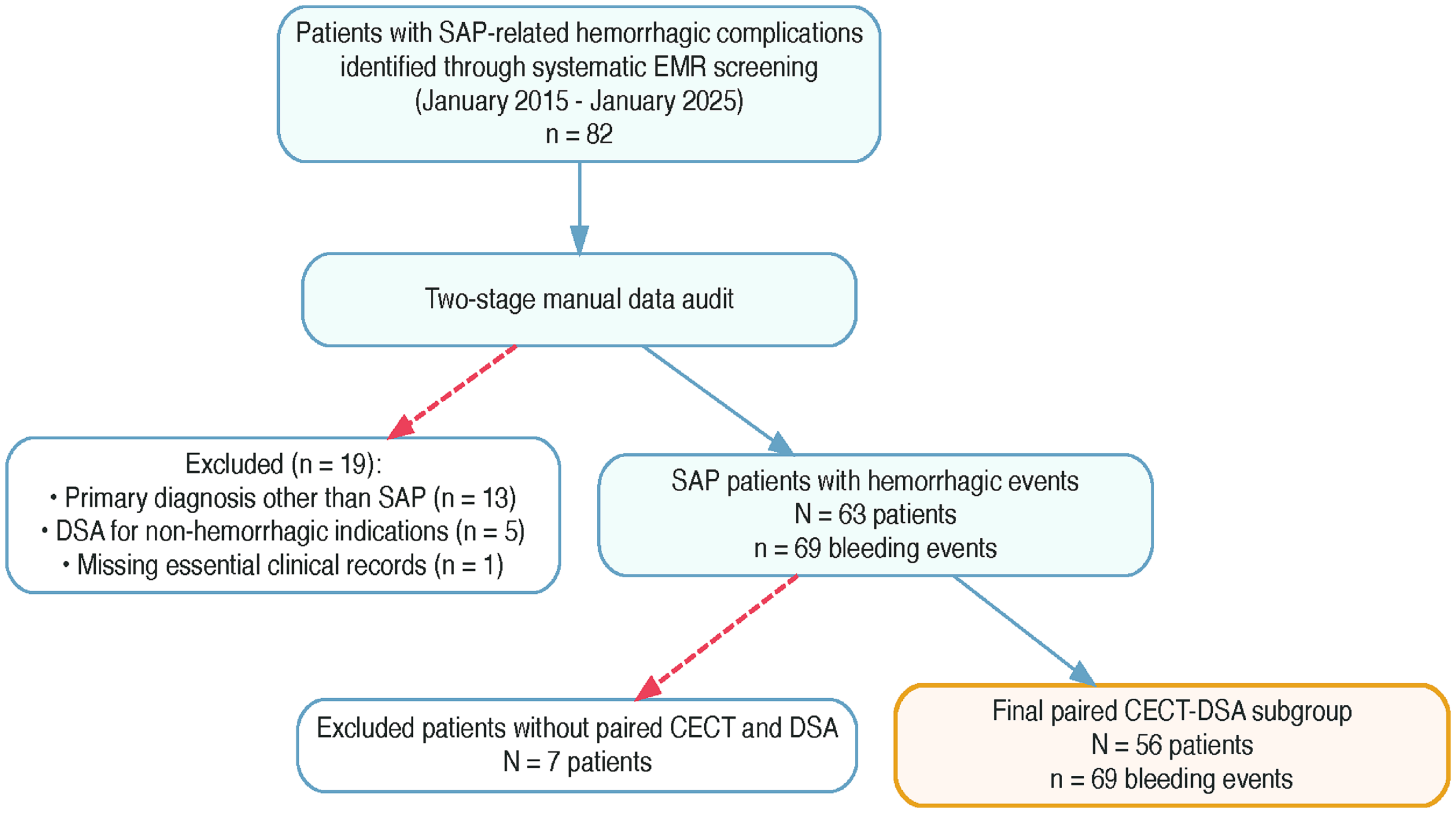
Flowchart of patient selection and inclusion in the study. Patients with severe acute pancreatitis (SAP) and suspected hemorrhagic complications were identified through systematic screening of the institutional database. After applying predefined inclusion and exclusion criteria and excluding cases without both imaging modalities or with incomplete data, 56 patients with 69 bleeding episodes who underwent both contrast-enhanced computed tomography (CECT) and digital subtraction angiography (DSA) were included in the final analysis. Reasons for exclusion are detailed in the flowchart.

A total of 82 patients with SAP and suspected hemorrhagic complications were initially screened from the institutional database. After applying the predefined inclusion and exclusion criteria, 56 patients with 69 bleeding episodes who underwent both CECT and DSA were included in the final analysis. The patient selection process is summarized in Figure 1.

### Patient-level and event-level characteristics

Baseline patient-level and event-level characteristics are summarized in Table 1. A total of 56 patients contributed 69 bleeding events. The cohort comprised 33 men (59%) and 23 women (41%), with a median age of 54 years (IQR 45–66 years). Biliary pancreatitis was the most common etiology (39%), followed by hypertriglyceridemia-induced (21%) and idiopathic causes (25%). Alcohol-related pancreatitis accounted for only a small proportion of cases (included within the Others category, 14%), in contrast to the etiological distribution typically reported in Western cohorts.

**Table 1:** Baseline characteristics and clinical presentations of bleeding events in the study population.

Statistics	Characteristics
Patient-level characteristics (*N* = 56)	
Gender (%)	
Male	33 (59%)
Female	23 (41%)
Age, years	54 (45, 66)
Etiology	
Biliary	22 (39%)
Hypertriglyceridemia-induced	12 (21%)
Idiopathic	14 (25%)
Others	8 (14%)
Bleeding frequency (%)	
1	33 (59%)
≥2	23 (41%)
Clinical outcome	
In-hospital mortality	29 (52%)
Survival	27 (48%)
Length of hospital stay, days	71 (40, 127)
Event-level characteristics (*n* = 69)	
Time from pancreatitis onset to bleeding, days	47 (34, 67)
Infected pancreatic necrosis at bleeding, *n* (%)	64 (93%)
Clinical presentation at bleeding, *n* (%)	
Abdominal pain	5 (7.2%)
Bloody drainage	50 (72%)
Combined drainage and GI bleeding	3 (4.3%)
Decrease in hemoglobin level	4 (5.8%)
Detected on routine CT follow-up	1 (1.4%)
Gastrointestinal bleeding	6 (8.7%)
Percutaneous drainage before bleeding	58 (84%)
Pancreatitis-related surgery before bleeding	43 (63%)

Data are presented as *n* (%) for categorical variables and median (interquartile range) for continuous variables. CT, computed tomography; GI, gastrointestinal; IPN, infected pancreatic necrosis; N/n, number of patients or events.

Thirty-three patients (59%) experienced a single bleeding episode, whereas 23 (41%) had recurrent bleeding. In-hospital mortality occurred in 29 patients (52%), and the median length of hospital stay was 71 days (IQR 40–127 days).

At the event level (*n* = 69), the median time from pancreatitis onset to bleeding was 47 days (IQR: 34–67 days), and the majority of bleeding events occurred after prior step-up interventions (PCD in 84%, pancreatitis-related surgery in 63%), reflecting the typically late, infected phase in which hemorrhagic complications emerge.

### Agreement between CECT grading and DSA grading

Diagnostic performance analysis was performed at the bleeding-event level. The distribution of CECT and DSA Grades is summarized in Table 2. On independent review, CECT findings were graded as Grade 0 in 26 (38%), Grade 1 in seven (10%), and Grade 2 in 36 (52%) events. DSA findings were graded as Grade 0 in 14 (20%), Grade 1 in four (6%), and Grade 2 in 51 (74%) events.

**Table 2:** Agreement between CECT grading and DSA grading for detection of bleeding in severe acute pancreatitis.

CECT Grade	DSA Grade 0	DSA Grade 1	DSA Grade 2	Total
CECT Grade 0	8	2	16	26
CECT Grade 1	4	0	3	7
CECT Grade 2	2	2	32	36
Total	14	4	51	69

Data are presented as number of bleeding events (percentage of total events). CECT Grades: Grade 0, Negative; Grade 1, Suspicious; Grade 2, Definite. DSA Grades: Grade 0, Negative; Grade 1, Indirect; Grade 2, Definite. Percentages are calculated based on the total number of events (*n* = 69); CECT, contrast-enhanced computed tomography; DSA, digital subtraction angiography.

Cross-tabulation revealed frequent discordance between modalities. Among 26 CECT Grade 0 events, 16 (62%) were classified as DSA Grade 2, indicating angiographically definite bleeding despite the absence of definite CT findings. Conversely, 4 of 36 CECT Grade 2 events (11%) were graded as DSA Grade 0–1. Overall intermodality agreement was limited, with a weighted Cohen *κ* of 0.29, corresponding to fair agreement.

### Diagnostic performance of CECT

Diagnostic performance metrics of CECT under different positivity thresholds are summarized in Table 3. Under the strict definition (Grade 2 vs. Grade 0, excluding Grade 1), CECT demonstrated a sensitivity of 66.7% and specificity of 80.0%. When indeterminate findings (Grade 1) were considered positive, sensitivity remained similar (67.3%), whereas specificity decreased to 57.1%. Conversely, when Grade 1 findings were grouped with negative examinations, sensitivity decreased modestly to 62.7%, while specificity increased to 77.8%. Across definitions, sensitivity ranged from 62.7 to 67.3%, whereas specificity ranged from 57.1 to 80.0%.

**Table 3:** Diagnostic performance of CECT using DSA as the reference standard according to different positivity definitions.

Positivity definition (CECT vs. DSA reference)	*n*	Sensitivity (%)	Specificity (%)	PPV (%)	NPV (%)
Strict: CECT Grade 2 vs. Grade 0 (excluding Grade 1)	58	66.7	80.0	94.1	33.3
Inclusive: CECT Grade 1–2 positive vs. Grade 0	69	67.3	57.1	86.0	30.8
Conservative: CECT Grade 2 positive vs. Grade 0–1	69	62.7	77.8	88.9	42.4

Diagnostic performance of CECT was calculated using DSA as the reference standard. CECT Grades: Grade 0, Negative; Grade 1, Suspicious; Grade 2, Definite. DSA Grades: Grade 0, Negative; Grade 1, Indirect; Grade 2, Definite. Three positivity definitions were applied: (i) strict definition (CECT Grade 2 positive vs CECT Grade 0 negative, excluding Grade 1); (ii) inclusive definition (CECT Grade 1–2 considered positive); and (iii) conservative definition (CECT Grade 2 considered positive and CECT Grade 0–1 negative). CECT, contrast-enhanced computed tomography; DSA, digital subtraction angiography; PPV, positive predictive value; NPV, negative predictive value.

### Clinical outcomes of DSA intervention

**Figure 2:**
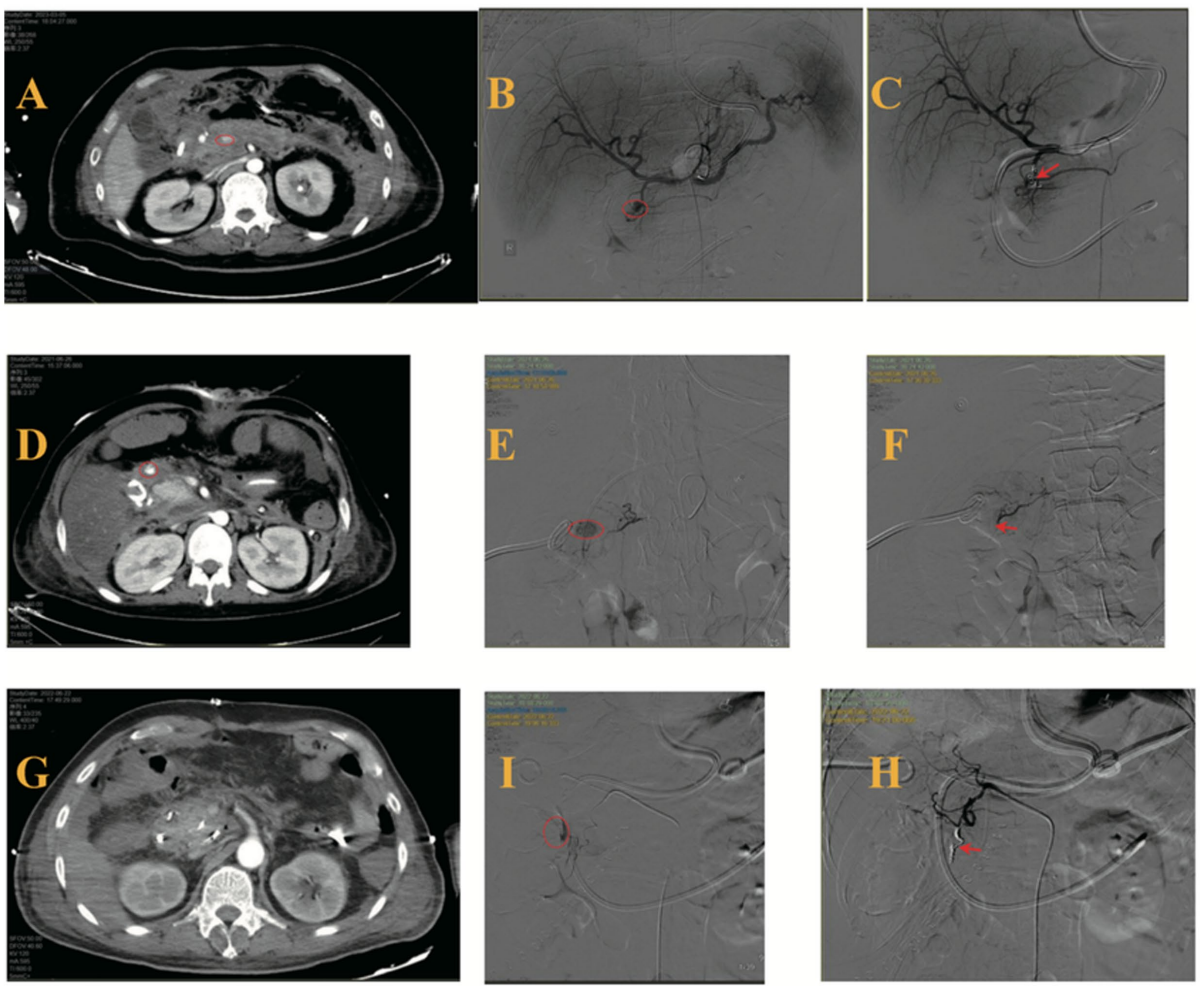
Representative imaging of severe acute pancreatitis (SAP)-associated hemorrhagic complications managed with digital subtraction angiography (DSA) and transcatheter arterial embolization. Red dashed circles indicate the bleeding sites, and red arrows indicate the embolization positions. Case 1: A 34-year-old man with idiopathic SAP developed intra-abdominal hemorrhage on day 63 after disease onset. **(A)** Contrast-enhanced CT (CECT) demonstrates a newly developed pseudoaneurysm arising from the gastroduodenal artery (GDA). **(B)** DSA confirms the pseudoaneurysm with active bleeding. **(C)** Post-embolization angiography demonstrates complete exclusion of the pseudoaneurysm without residual contrast extravasation. Case 2: A 22-year-old man with hypertriglyceridemia-associated SAP developed intra-abdominal hemorrhage approximately 2 months after distal pancreatectomy, splenectomy, and external drainage. **(D)** CECT demonstrates active hemorrhage adjacent to the duodenum. **(E)** Selective angiography of the superior mesenteric artery reveals persistent active contrast extravasation from a branch of the pancreaticoduodenal artery. **(F)** Post-embolization angiography confirms complete cessation of contrast extravasation following embolization with 100–300 μm gelatin sponge particles. Case 3: A 77-year-old man with biliary pancreatitis developed intra-abdominal hemorrhage during the postoperative period following surgical drainage for infected pancreatic necrosis complicated by severe retroperitoneal inflammation, gastrointestinal fistula formation, and temporary loop ileostomy. **(G)** CECT demonstrated no definite active contrast extravasation or newly formed hematoma. **(H)** Despite negative CECT findings, persistent clinical suspicion prompted DSA, which revealed active bleeding from a distal branch of the gastroduodenal artery (GDA). **(I)** Post-embolization angiography demonstrated successful hemostasis with complete occlusion of the culprit vessel after coil embolization.

The clinical outcomes following DSA intervention are detailed in Table 4. Overall, technical and clinical success (Result 1) was achieved in 72.5% (50/69) of cases. Among 26 events with CECT Grade 0, 69.2% (18/26) resulted in successful therapeutic embolization (Result 1), whereas only 23.1% (6/26) were confirmed as truly negative and stable (Result 3). In contrast, for CECT Grade 2 events, the rate of therapeutic intervention (Result 1 and 2) reached 91.7% (33/36). All instances of rebleeding (Result 2, *n* = 4) and procedural abandonment (Result 4, *n* = 1) occurred in the Grade 2 group. Representative paired CECT and DSA imaging findings from three hemorrhagic events are shown in Figure 2.

**Table 4:** Distribution of DSA intervention outcomes across different CECT grades during hemorrhagic episodes (*n* = 69 events).

CECT Grade	Result 0 (Neg-Active)	Result 1 (Embo-Success)	Result 2 (Embo-Rebleed)	Result 3 (Neg-Stable)	Result 4 (Abandoned)	Total
Grade 0	2 (7.7%)	18 (69%)	0 (0%)	6 (23%)	0 (0%)	26 (100%)
Grade 1	1 (14%)	3 (43%)	0 (0%)	3 (43%)	0 (0%)	7 (100%)
Grade 2	2 (5.6%)	29 (81%)	4 (11%)	0 (0%)	1 (2.8%)	36 (100%)
Total	5 (7.2%)	50 (72%)	4 (5.8%)	9 (13%)	1 (1.4%)	69 (100%)

Data are presented as *n* (%). Percentages are calculated based on the total number of events within each CECT grade. CECT Grade: Grade 0 (Negative), Grade 1 (Suspicious), and Grade 2 (Definite). DSA Outcome: Result 0 (Neg-Active), negative angiography with clinically suspected persistent hemorrhage; Result 1 (Embo-Success), successful control via embolization; Result 2 (Embo-Rebleed), rebleeding after technical success; Result 3 (Neg-Stable), negative findings with subsequent clinical stabilization; Result 4 (Abandoned), procedure terminated due to instability. CECT, contrast-enhanced computed tomography; DSA, digital subtraction angiography.

The single case (1.4%) was categorized as Result 4 (Abandoned). This involved a patient with a delayed diagnosis of retroperitoneal hemorrhage who exhibited extreme hemodynamic instability during the procedure. The intervention was prematurely terminated as the patient’s physiological status precluded completion of DSA; subsequently, the family opted for comfort care and declined further surgical salvage.

### Surgical outcomes following DSA intervention

Of the 69 events, nine (13.0%) required surgical intervention following DSA, including five after Result 0 (Neg-Active) and four after Result 2 (Embo-Rebleed). Definitive hemostasis was achieved in all five R0-triggered cases, compared with one of the four Result 2-triggered cases. These surgical outcomes were reported descriptively to characterize the complete clinical pathway beyond DSA in this cohort; formal surgical outcome classification was not a pre-specified endpoint of this study. These data were included to provide a more comprehensive view of the downstream clinical trajectory following failed or inconclusive endovascular management.

## Discussion

In this study comparing CECT with DSA in SAP-associated hemorrhagic complications, we observed only fair intermodality agreement and a consistent tendency of CT to underestimate angiographically confirmed bleeding. Nearly 70% of cases with negative CT findings were subsequently confirmed to have active bleeding requiring therapeutic embolization. To our knowledge, this is one of the few studies to quantitatively demonstrate the high rate of clinically significant, angiographically confirmed bleeding in CT-negative SAP patients, and the results expose a critical limitation of relying solely on CT to exclude hemorrhage in this high-risk population.

Several technical and biological factors converge to produce this diagnostic gap. The corrosive enzymatic environment in SAP frequently generates intermittent or low-flow arterial bleeding that escapes detection during a single contrast-enhanced acquisition, particularly within complex necrotic collections and evolving vascular injury (7, 17). This is frequently compounded by complex retroperitoneal anatomy where extensive necrosis and inflammatory exudates obscure subtle vascular abnormalities (5, 18). Additionally, the limited spatial resolution of CECT relative to selective, real-time catheter angiography often precludes the detection of low-flow venous leaks or micro-pseudoaneurysms (12, 18). By contrast, selective catheter angiography enables real-time dynamic assessment and targeted vessel interrogation, facilitating identification of subtle lesions invisible on cross-sectional imaging (17, 19). The higher angiographic detection rate observed in this study supports the continued role of DSA as both a diagnostic and therapeutic modality in this setting (6, 18).

Notably, the diagnostic performance of CT is threshold dependent. Although definite CT findings (Grade 2) demonstrated a high positive predictive value for angiographically confirmed bleeding, CT-negative examinations did not reliably exclude DSA-proven hemorrhage (12, 17). For interventional radiologists, this underscores the fact that reliance solely on the absence of CT extravasation may delay the necessary angiographic evaluation in clinically suspected cases (12, 17).

These results do not diminish the value of CT as an initial tool for triage. Rather, they suggest that CT and DSA provide complementary information and that angiographic evaluation should be considered when clinical suspicion persists despite negative CT findings (1, 18). The graded framework applied in this study may facilitate more standardized communication between diagnostic and interventional teams.

Our findings show a significant correlation between CECT grading and long-term success of endovascular treatment. Patients with negative or subtle CT signs (Grade 0/1) typically achieved stable results after DSA, whereas those with definite extravasation on CT (Grade 2) were more likely to experience rebleeding despite initial technical success. This difference likely reflects the progression of vascular injury in SAP. In Grade 0/1 cases, the blood vessel structure is relatively preserved, providing a better site for embolic agents before major vessel disruption occurs. In contrast, Grade 2 presentation suggests severe vessel wall erosion (7, 17). At this advanced stage, intense local inflammation may continue to damage the treated vessel, leading to recurrent bleeding (17).

Two partially overlapping mechanisms warrant separate consideration when interpreting hemorrhage in this cohort. Percutaneous drainage and surgical necrosectomy may directly injure adjacent vessels or subject them to mechanical stress, thereby contributing to procedure-related bleeding (4, 5, 7). Concurrently, infected pancreatic necrosis—present in 93% of bleeding events in our series—drives a sustained proteolytic and inflammatory process that can erode arterial walls independently of iatrogenic injury, frequently resulting in pseudoaneurysm formation in vessels remote from the intervention site (1, 5, 18). The high prevalence of prior PCD or surgery in our cohort should therefore not be interpreted as evidence that bleeding was predominantly procedure-induced; rather, it reflects the advanced infected disease stage in which both invasive interventions and hemorrhagic complications commonly occur. Disentangling these mechanisms in individual cases remains challenging and warrants further prospective investigation (20).

The success of hemostasis also depends significantly on the local vascular anatomy. Complex collateral circulation in the abdomen poses a major challenge for intervention; if the vessel shape prevents complete isolation of the lesion using the ‘sandwich’ technique, back-flow from collaterals can quickly cause rebleeding (5, 17, 21). Additionally, new bleeding often occurs from fresh pseudoaneurysms or ruptures in previously healthy arteries exposed to corrosive fluids due to extensive pancreatic necrosis (6, 17).

Taken together, our findings suggest that, in this specific high-risk population, persistent clinical signs of bleeding—most commonly bloody drainage or an unexplained hemoglobin drop—may justify further angiographic evaluation even when CECT findings are negative or indeterminate (12, 22). We do not propose that clinical judgment should replace cross-sectional imaging; rather, the two should be interpreted in an integrated manner, with the threshold for proceeding to DSA individualized according to clinical context, hemodynamic status, and institutional resources (18, 23). Owing to the retrospective design and the inherent selection bias of including only patients who underwent both CECT and DSA, these observations should be regarded as hypothesis-generating rather than definitive practice recommendations.

**Figure 3:**
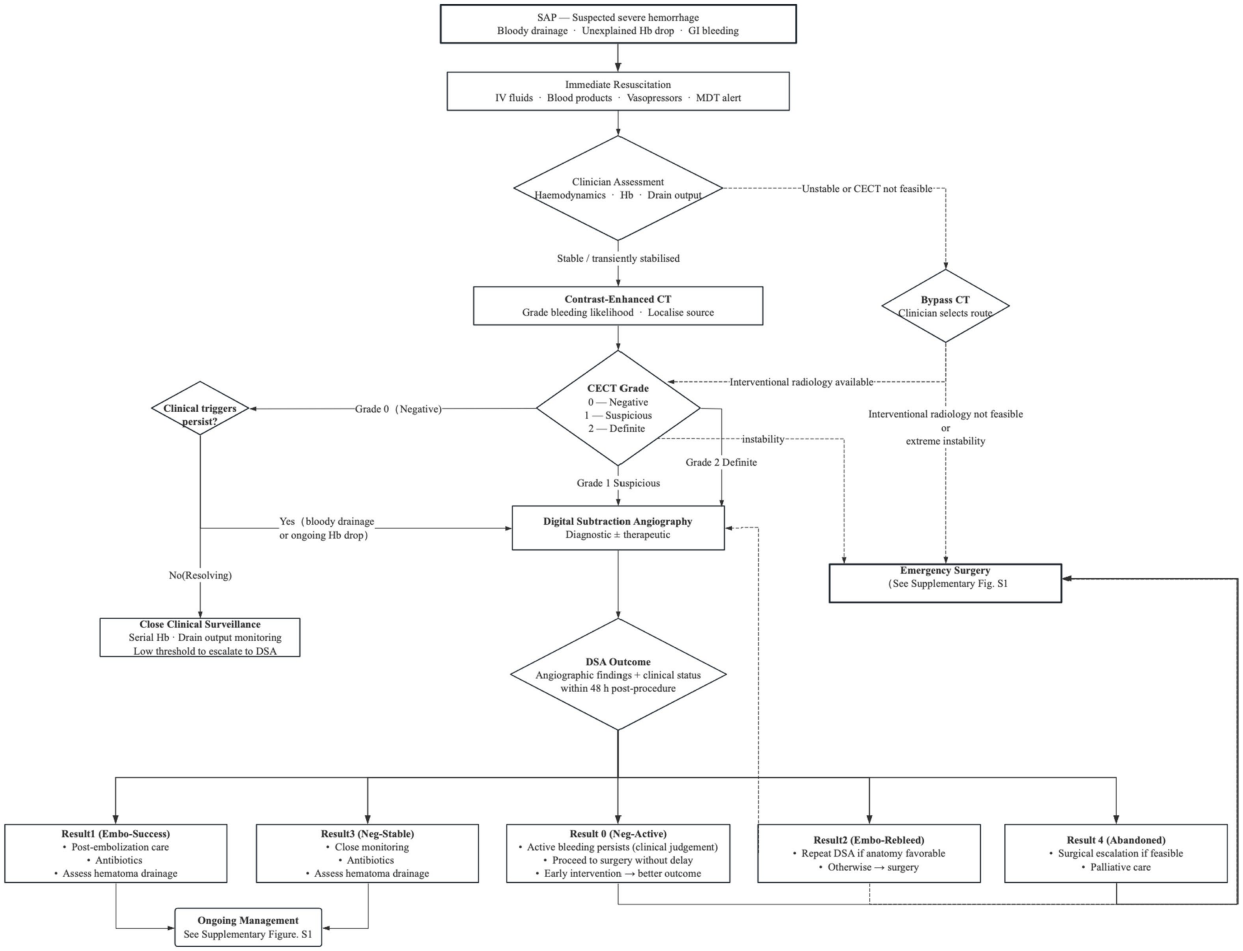
Proposed management algorithm for severe hemorrhage in acute pancreatitis informed by single-center real-world data. Hemodynamically unstable patients or those in whom CT is not feasible may bypass imaging and proceed directly to DSA (if interventional radiology is immediately available) or to emergency surgery. For stable patients, CECT was performed, and the findings were graded as follows: Grade 0, Negative (absence of contrast extravasation, pseudoaneurysm, or progressive hematoma); Grade 1, Suspicious (atypical high-density foci, enlarging hematoma without clear extravasation, or suspicious vascular abnormalities); Grade 2, Definite (clear contrast extravasation, definitive pseudoaneurysm, or signs of active hemorrhage). Grade 0 findings with resolving clinical symptoms were managed with close clinical surveillance; Grade 0 with persistent clinical triggers (bloody drainage or ongoing Hb drop), Grade 1, and Grade 2 findings proceeded to DSA. DSA outcomes were classified based on angiographic findings and clinical status within 48 h post-procedure: Result 0 (Neg-Active), negative angiography with clinically suspected persistent hemorrhage; Result 1 (Embo-Success), successful hemostatic control via embolization; Result 2 (Embo-Rebleed), rebleeding after technical success; Result 3 (Neg-Stable), negative findings with subsequent clinical stabilization; Result 4 (Abandoned), procedure terminated due to instability. Result 1 and Result 3 proceeded to ongoing management, whereas Result 0, Result 2, and Result 4 required surgical escalation or emergency surgery. The complete surgical escalation and post-intervention surveillance pathways are presented in Supplementary Figure S1. All escalation decisions require assessment by an experienced clinician; imaging findings are adjuncts to, not substitutes for, clinical judgment. CECT, contrast-enhanced computed tomography; DSA, digital subtraction angiography; Hb, hemoglobin; MDT, multidisciplinary team; SAP, severe acute pancreatitis.

Based on the findings of this study, we describe a single-center decision-support framework for SAP-related hemorrhage. This algorithm is intended to support, but not to replace, multidisciplinary clinical judgment, and its external validity remains to be established in larger, prospective, multicenter cohorts (Figure 3). The complete pathway, including surgical escalation and post-intervention surveillance, is provided in Supplementary Figure S1.

An additional observation worth noting is the etiological distribution of our cohort, in which biliary and hypertriglyceridemia-induced pancreatitis predominated, while alcohol-related pancreatitis was rarely encountered. This pattern contrasts with many Western series, in which alcohol consistently ranks among the leading causes of SAP but is consistent with epidemiological trends recently reported across eastern China, where biliary disease remains the dominant etiology and hypertriglyceridemia-associated pancreatitis has risen markedly over the past decade in parallel with changes in dietary habits and metabolic disease prevalence (24, 25). The vascular complications central to this study—pseudoaneurysm formation, arterial erosion, and active extravasation—arise primarily from the proteolytic and inflammatory destruction of vessel walls and are not thought to be etiology-specific (1, 4).

The in-hospital mortality observed in our cohort was 52%, which is substantially higher than the 15–30% typically reported for unselected SAP populations (23). Several factors likely contribute to this disparity. Our study was deliberately restricted to patients who developed clinically suspected major hemorrhage and underwent both CECT and DSA—an inherently high-risk subset representing the most severe end of the SAP spectrum. Infected pancreatic necrosis was present in 93% of bleeding events, and most patients had already failed conservative management and undergone step-up interventions, indicating an advanced and highly complicated disease course (26, 27). Recurrent bleeding occurred in 41% of patients, and rebleeding after initially successful embolization is well recognized as a strong predictor of mortality in this setting (6). The high mortality, therefore, reflects the profound severity of the underlying disease and the selection bias of a high-risk cohort, rather than a direct consequence of DSA or embolization itself (21). Whether bleeding episodes and the need for endovascular or surgical intervention independently contribute to mortality could not be formally assessed in this descriptive analysis and warrants dedicated investigation in future studies.

## Limitations

This study has several limitations. As a retrospective single-center analysis restricted to bleeding events in which both CECT and DSA were performed, our cohort represents a selected, severely ill subset of SAP patients, and the resulting referral and verification bias is also likely to account for the high in-hospital mortality observed (15, 17, 21); patients with negative CT findings and no ongoing clinical suspicion of bleeding did not proceed to angiography and were therefore not captured. Bleeding events rather than individual patients were used as the unit of analysis, which may introduce inter-event correlation, and although the interval between CECT and DSA was generally limited to a few hours when clinically feasible, it was not uniformly recorded, so temporal mismatch may have contributed to the discordance observed between modalities. DSA was used as the reference standard for practical reasons, but it is not an absolute ground truth—it is insensitive to venous and low-flow bleeding, may miss intermittent or spontaneously resolved hemorrhage, and remains operator-dependent (1, 5, 22)—and the diagnostic metrics reported here should accordingly be read as agreement against an imperfect comparator. Finally, our cohort was dominated by biliary and hypertriglyceridemia-induced pancreatitis, with very few alcohol-related cases, reflecting the epidemiology of SAP in eastern China and limiting the direct generalizability of these findings to populations in which alcohol plays a larger etiological role.

## Conclusion

In this selected high-risk cohort of patients with severe acute pancreatitis undergoing both CECT and DSA for clinically suspected hemorrhagic complications, CECT demonstrated only limited concordance with angiographic findings and frequently underestimated angiographically confirmed bleeding. Negative or non-definitive CT findings should therefore be interpreted cautiously when clinical suspicion for ongoing hemorrhage persists, and angiographic evaluation should be considered as part of an integrated, clinically driven diagnostic and therapeutic pathway in this population. CECT and DSA provide complementary information, and a multidisciplinary, clinically anchored decision-making framework—of which the management algorithm proposed in this study represents one practical example—may help optimize the timing and selection of interventional management in SAP-associated hemorrhage. Prospective multicenter validation is warranted to refine decision thresholds and to externally validate the proposed pathway.

## Data Availability

The raw data supporting the conclusions of this article will be made available by the authors, without undue reservation.
