# Supplementary material for: Discordance between CECT and angiographic findings in severe acute pancreatitis-related hemorrhage: implications for interventional management
Source: Front Med (Lausanne). 2026 Jun 1;13:1845142. doi: 10.3389/fmed.2026.1845142 (PMC13265546; doi:10.3389/fmed.2026.1845142)
Supplement: Supplementary file 1 [file Table_1.docx]

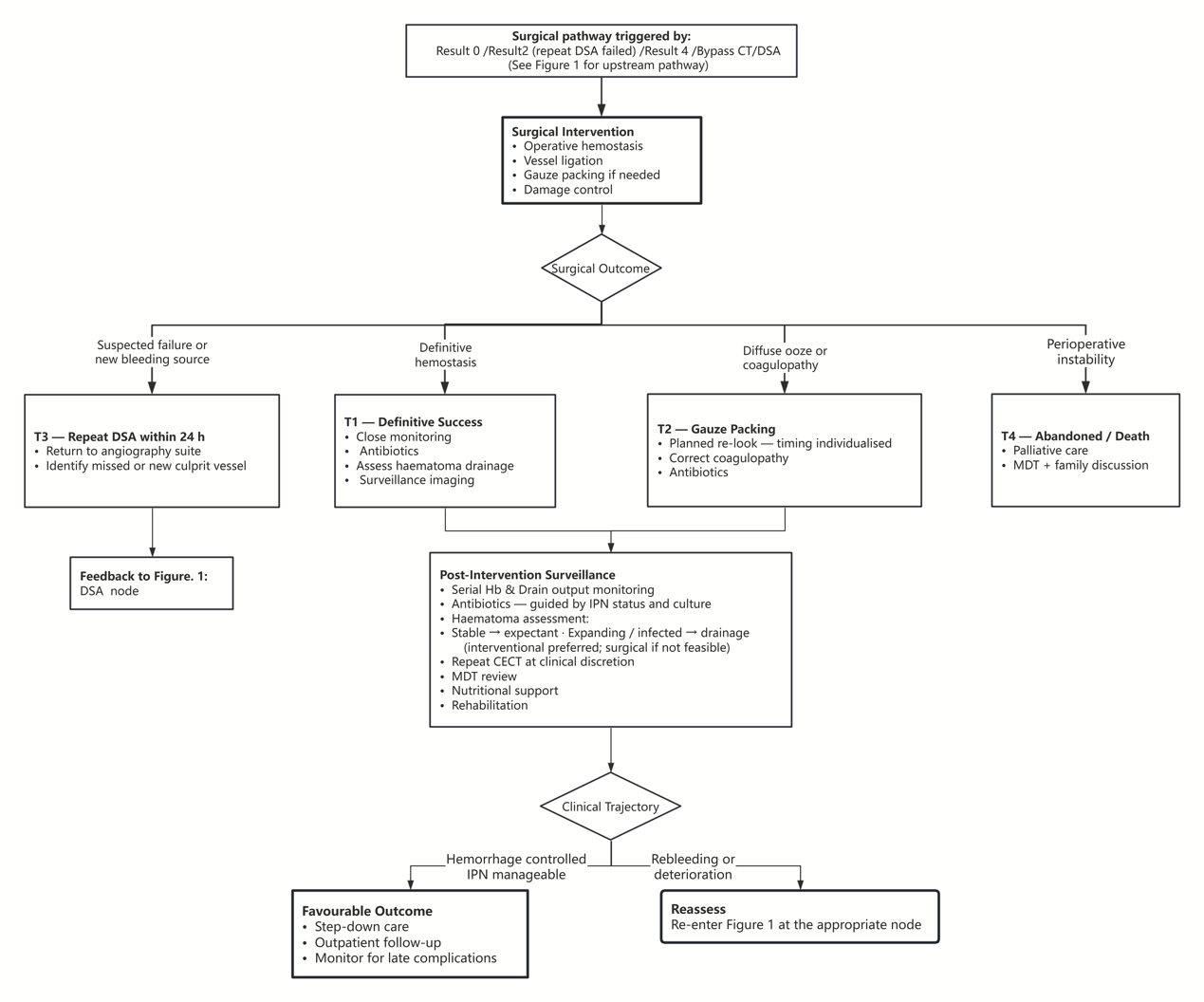


**Supplementary Figure S1. Surgical escalation and post-intervention surveillance pathway for hemorrhagic complications in severe acute pancreatitis.**

This figure depicts the management pathway for patients requiring surgical intervention following DSA (Result0, Result 2, or Result 4) or those who bypass CT/DSA due to hemodynamic instability (see Figure 1 for the upstream decision pathway). Surgical outcomes are classified as: T1 (Definitive Success) , operative hemostasis achieved; T2 (Gauze Packing) , hemostasis incomplete, gauze packing applied with planned re-look, timing individualized according to physiological recovery and coagulopathy correction; T3 (Repeat DSA within 24 h) , suspected surgical failure or new bleeding source identified, returning to Figure 1 DSA node; T4 (Abandoned/Death) , procedure terminated due to perioperative instability or death. T1 and T2 proceed to post-intervention surveillance, which encompasses serial Hb and drain output monitoring, antibiotic therapy guided by infected pancreatic necrosis (IPN) status and culture results, hematoma assessment (stable: expectant management; expanding or infected: drainage, with interventional approach preferred), repeat CECT at clinical discretion, MDT review, nutritional support, and rehabilitation. Patients with rebleeding or clinical deterioration re-enter Figure 1 at the appropriate decision node. **Abbreviations:** CECT, contrast-enhanced computed tomography; DSA, digital subtraction angiography; Hb, hemoglobin; IPN, infected pancreatic necrosis; MDT, multidisciplinary team; SAP, severe acute pancreatitis.
